# Anomalous Left Superior Vena Cava Predisposing to Recurrent Brain Abscesses: A Case Report and Review of the Literature

**DOI:** 10.7759/cureus.62329

**Published:** 2024-06-13

**Authors:** Adnan Shaik, Karan Joseph, Rahim Abo Kasem, Angela Downes, Muhammed B Janjua

**Affiliations:** 1 Neurosurgery, University of Missouri Kansas City School of Medicine, Kansas City, USA; 2 Neurosurgery, Washington University School of Medicine, St. Louis, USA; 3 Neurosurgery, Medical University of South Carolina, Charleston, USA; 4 Neurosurgery, University of Colorado Anschutz Medical Campus, Aurora, USA

**Keywords:** pulmonary circulation, right-left shunt, plsvc, recurrent brain abscess, anomalous left superior vena cava

## Abstract

Brain abscess is a devastating illness, with a high risk of morbidity and mortality. Recurrent brain abscess poses a challenge to diagnosis while treatment options may differ. Right to left shunt is a lesser-explored etiology for recurrent brain abscesses. PubMed literature review was performed to study all published studies with reference to right to left cardiac shunt as a possible etiology for the recurrent brain abscesses. The authors also report a case of a young male who developed recurrent brain abscess after previous resection and treatment. Right to left cardiac shunting of deoxygenated blood is an etiology for recurrent brain abscess formation. Thorough systemic workup and multispecialty treatment is recommended to treat this relatively uncommon presentation.

## Introduction

The management of brain abscesses is a major challenge in neurosurgery and poses a significant healthcare burden. Despite recent advancements in antibiotic therapy and imaging modalities, brain abscess requires a high index of clinical suspicion and collaborative input from many members of the healthcare team to reduce associated morbidity and mortality. The presentation has been described as a classic triad of headache, fever, and focal neurologic deficits; patients who present with this triad should be worked up with immediate neuroaxis imaging to look for brain abscess [1].

Contiguous spread from infections such as sinus infections and otitis media or following surgery are the most common etiologies for the formation of brain abscesses [2-3]. Hematogenous spread is also a common cause and may be associated with up to one-third of these cases [4]. If hematogenous spread is suspected, it is important to make every possible effort to identify a primary source of infection. At times, these infections can be occult and may need multisystem imaging for diagnosis. In the setting of recurrent brain abscess, the existence of a right-to-left shunt should be strongly considered in the etiology differential. Pulmonary arterial venous malformations (PAVM) and anomalous venous structures along with septal deformities like patent foramen ovale are examples of anatomical variants that may result in venous blood bypassing the pulmonary vasculature and predisposing patients to intracranial infections [4]. The most common thoracic venous anomaly is a persistent left superior vena cava (PLSVC), which may rarely drain into the left atrium, creating a right to left shunt, predisposing the patient to systemic seeding of an occult infection [5]. We present a case of a male patient with recurrent brain abscesses who was found to have a PLSVC with drainage of venous blood into the left atrium.

## Case presentation

**Figure 1:**
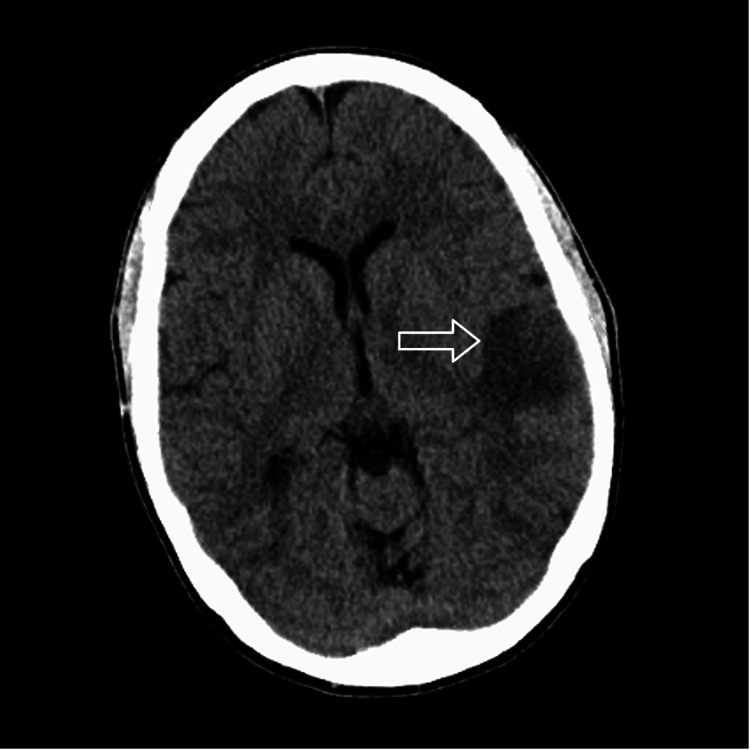
Head CT scan without contrast revealing subcortical hypodensity in the left temporal region with adjoining cortical sulci effacement.

**Figure 2:**
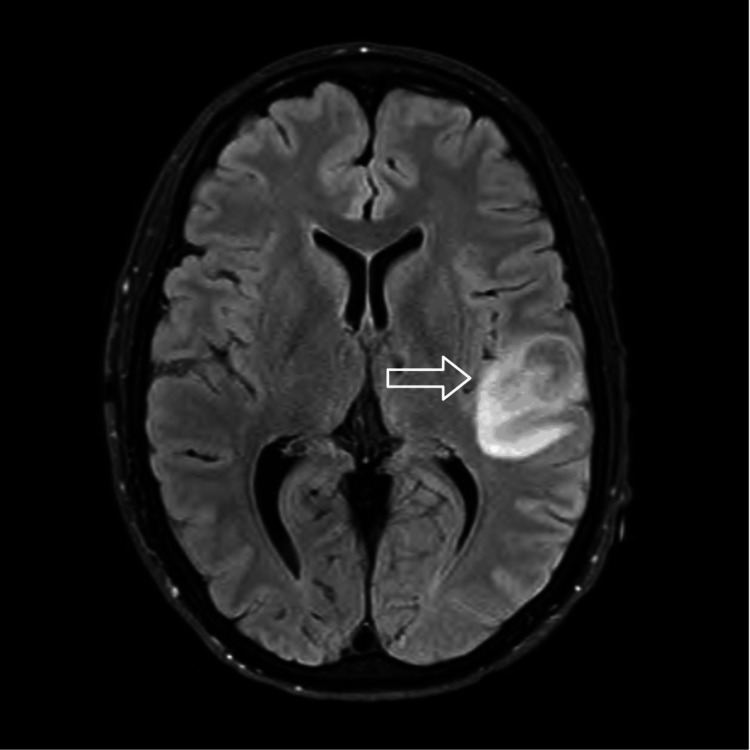
MRI brain scan with and without contrast with axial T2/FLAIR sequence revealing 17 x 20 mm round/oblong lesion with peripheral T2 hyperintensity centered around the superior temporal gyrus.

**Figure 3:**
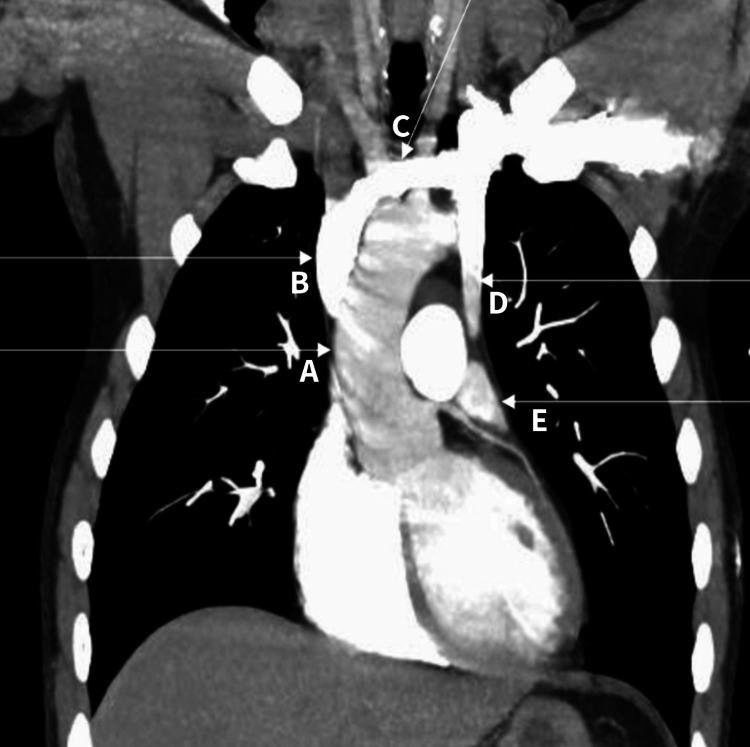
CT pulmonary angiography shows an anomalous persistent left superior vena cava. CT pulmonary angiography revealed a normal superior vena cava (B) and aorta (A). An anomalous persistent left superior vena cava (D) arising from the left innominate vein (C) and draining into the left atrium (E) is evident.

A 39-year-old male with a past medical history of brain abscess diagnosed at the age of six, presented to the emergency department (ED) with a history of multiple seizures, and a recent grand mal seizure resulting in a violent fall. On admission, the patient suffered from epistaxis, overt symptoms of lethargy, aphasia, and dysarthria, and was uncooperative with the neurological examination. Collateral information obtained from a family member revealed that the patient had experienced headaches for the past three days with low-grade fevers. A comprehensive review of systems was otherwise negative. A review of past medical history revealed a right parietal brain abscess which resulted in a formal large right-sided craniotomy at the age of 6. Neurological examination was consistent with symptoms of clouding of the sensorium with receptive aphasia. The patient was uncooperative for the detailed cerebellar examination and motor strength examination but was able to move against gravity in all extremities. Intravenous (IV) Fosphenytoin was administered in the ED. A head CT scan without contrast revealed hypodensity in the left superior temporal lobe and a facial bone fracture (Figure 1). MRI brain both with and without contrast confirmed a 17 x 20 mm oblong lesion with peripheral ring enhancement centered around the left superior temporal gyrus, which was concerning for a brain abscess (Figure 2). After multispecialty and formal discussion with the patient and family, evacuation of the brain abscess with a craniotomy was recommended. Surgery was performed, including left temporal craniotomy, and an inverted U-shaped durotomy was performed. The transsulcal approach was utilized for access to the left superior temporal gyrus lesion. After samples were taken for gram stain culture and sensitivity, formal evacuation of purulent material was achieved. The drainage site was washed with a copious amount of normal saline irrigation solution. The patient was started on intravenous antibiotics comprising Cefepime and Vancomycin. Metronidazole was added when gram stain and cultures revealed the microbial agent to be an anaerobic gram-negative rod, including Fusobacterium nucleatum and Campylobacter rectus. Due to the prior history of right parietal brain abscess, a thorough multisystem evaluation and workup was performed. A carotid ultrasound, maxillofacial CT scan, and a transesophageal echocardiogram revealed no source of infection or right-to-left shunt. However, computed tomography pulmonary angiogram (CTPA/CTPE) revealed an anomalous venous structure arising from the left subclavian-brachiocephalic juncture and draining into the left atrium, which was consistent with persistent left superior vena cava (PLSVC) (Figure 3). Interventional Cardiology was consulted and right and left heart catheterization was performed to ensure that the anomalous vessel was not essential for hemodynamic stability and cardiac flow, as well as to rule out any coexisting coronary artery disease given the patient’s long-standing smoking history. With these concerns addressed, the patient underwent open thoracotomy and ligation of the anomalous vessel. At six months follow-up, the patient was devoid of any active neurological or cardiac symptoms. The patient remained in good health and continued to make an optimal recovery.

## Discussion

Methods

PubMed Medline literature search was performed to study all published studies with reference to the right to left cardiac shunt as a possible etiology for recurrent brain abscesses. We also report a unique case of a young male who presented with recurrent brain abscesses and was found to have PLSVC. Published case studies were selected for detailed literature review and for further analysis. Student t-test and Mann-Whitney U test were used for the formal statistical analysis.

After a detailed literature review, 12 case reports were found that discussed right to left shunt as a possible etiology for recurrent brain abscess formation [6-17]. The oldest reported case among the 12 case reports was published in 1971 [16]. The data collected from our literature search is shown in Table 1. Eleven patients were identified for the final data analysis. The median age was 40, and eight out of the 11 patients were males. Two patients died of complications from the recurrent brain abscess [6,15]. One of the patients had a persistent left-sided superior vena cava and an absent right-sided vena cava [18].

**Table 1:** Patient characteristics of prior case reports with PLSVC and brain abscess formation. PLSVC: Persistent left superior vena cava

Case Report #	Year of publication	Age of patient (years)	Gender	Mortality	History of prior brain abscess	Hypoxemia/Cyanosis	Source
1	1971	5	Male	No	No	N/A	Sherafat et al., 1971 [16]
2	1985	46	Male	Yes	No	No	Schick et al., 1985 [15]
3	1986	14	Female	No	No	No	Looyenga et al., 1986 [10]
4	1989	27	Male	No	No	N/A (did not mention)	Fujimura et al., 1989 [17]
5	2005	46	Male	No	No	N/A	Troost et al., 2005 [14]
6	2006	12	Female	No	No	Yes (clinically mild)	Akkoyun et al., 2006 [11]
7	2009	40	Male	Yes	No	N/A	Ansari et al., 2009 [6]
8	2011	47	Female	No	No	No	Lee et al., 2011 [13]
9	2012	53	Female	No	Yes	Yes	Ch’ng et al., 2012 [8]
10	2013	58	Male	No	No	N/A (no mention)	Barba et al., 2013 [9]
11	2014	36	Male	No	Yes	Yes (hypoxemia)	Menachem et al., 2014 [7]
12	2018	49	Male	No	No	N/A (no mention)	Lloret-Villas et al., 2018 [12]

The authors also report a unique case of a 39-year-old male with a prior history of brain abscess status post-surgical drainage and resection of the parietal lobe at six years of age. The patient’s presented symptoms led to brain imaging and a formal diagnosis of recurrent brain abscess was made. Imaging was reviewed by the senior author (MBJ). After discussion with the infectious disease, a plan was made to drain the abscess for source control and identification of the organism. Therefore, a formal mini-craniotomy centered on the abscess was planned and the infected portion of the superior temporal lobe was carefully resected after drainage of the abscess. The patient was treated with an appropriate course of antibiotic therapy after the surgery. The patient made a remarkable recovery after surgery without any neurological deficits. The recurrence of his brain abscess warranted additional systemic workup which revealed a PLSVC. A multi-specialty approach was utilized to address this anomalous route of recurrent infection.

Brain abscess is a morbid infection of the brain parenchyma and can have a stuttering course if not treated promptly. Moreover, recurrent brain abscesses are relatively uncommon but demand a thorough systemic workup. Any anatomic variant that bypasses the pulmonary capillary beds is a risk factor for recurrent intracranial infections, including abscesses [16]. However, other abnormalities in which some blood passes from the venous system to systemic arteries without encountering pulmonary capillaries may entirely be asymptomatic except for an increased risk of paradoxical emboli, both infectious and non-infectious [19]. One such example where this may be the case is a pulmonary arteriovenous malformation (PAVM). In these malformations, blood flows through the low-resistance intrapulmonary fistulas, moving from pulmonary arteries to pulmonary veins without encountering capillaries. Depending on the degree of shunting, cyanosis and systemic hypoxemia may be present, but an increased risk of paradoxical emboli may be the only consequence in other cases [20]. Other right-to-left shunts arise from abnormalities in the embryological development of the systemic venous system, similar to the presented case.

PLSVC is found in 0.3-0.5% of the general population [5]. Most cases of PLSVC drain into the right atrium. In about 10% of cases, PLSVC drains into the left atrium causing a right-to-left shunt, as in the presented case [18]. An interesting observation in this case is the lack of hypoxia. When a PLSVC drains into the right atrium, patients remain asymptomatic and the PLSVC does not carry any major implications. On the other hand, when the PLSVC drains into the left atrium, hemodynamic instability and raised right atrial pressures can lead to hypoxia. Even though our patient’s PLSVC drained into the left atrium, our patient did not have any hypoxia. We hypothesize that the connection between the PLSVC and the right-sided superior vena cava via the innominate vein is the reason for normal oxygen levels in our patient.

There are two major veins in the human embryo that act as precursors to the superior and inferior vena cava. Superior and inferior cardinal veins will receive blood from caudal and cranial regions of the embryo, and drain back into the embryologic heart precursor. The right common cardinal vein along with the caudal aspect of the right superior cardinal vein will form the right superior vena cava. In typical embryologic development, the left superior cardinal vein along with the left common cardinal vein will regress. If they do not regress, then a PLSCV is formed. There is no established basis for understanding exactly why this phenomenon occurs, however, one prominent theory proposes that any anomaly that could result in reduced left atrial pressure would lead to stunted development of the left atrium. The subsequent stunted left atrium would not be able to compress the left cardinal veins, resulting in a persistent left common cardinal vein along with the caudal aspect of the superior cardinal vein [5].

PLSVCs lead to brain abscesses by bypassing the pulmonary circulation. Normally, bacteria found in the oral flora can enter the bloodstream during dental procedures and brushing teeth. However, the aerobic environment of the lungs and alveolar macrophages lead to the death of the anaerobic bacteria. As such, the pulmonary circulation serves as a filter, preventing anaerobic bacteria from entering the systemic circulation. In patients with PLSVC, a right-to-left shunt is created, hence bypassing the pulmonary circulation. As a result, anaerobic bacteria can bypass pulmonary circulation and seed into the brain [7-9]. The patient’s surgical cultures grew Fusobacterium nucleatum and Campylobacter rectus. Fusobacterium nucleatum and Campylobacter rectus are both anaerobic bacteria that are commonly associated with periodontitis. A prompt and thorough workup is needed to aid in early diagnosis. Imaging can guide toward the next step of surgical intervention for early source control and to aid in targeted antibiotic therapy.

There are some limitations of this study. The study design is retrospective, so the results are based on previously published studies. Associated selection bias from previously published case studies cannot be eradicated. The authors also failed to delineate the exact microbic details. The represented case is only one case and the authors have not observed any more patients of brain abscess with similar venous anomaly.

## Conclusions

Brain abscess is a devastating infection of brain parenchyma with significant morbidity. Recurrent brain abscesses pose a great challenge to treating physicians. The authors highlight a unique association that stands as the main etiology for recurrent brain abscesses in the presented case of a young male. Persistence of PLSVC creates an alternative passage of venous blood directly into the left atrium while creating a right-to-left shunt which increases the possibility of recurrent brain abscesses in a healthy individual in the absence of patent foramen ovale. The patient was treated with surgical drainage of the abscess and subsequently underwent ligation of the anomalous vessels after a thorough clinical cardiac workup. A multi-specialty approach is prudent to treat recurrent brain abscesses. Due to the rarity of this etiology, a randomized clinical study will be helpful to further validate this association.
